# Supplementary figures and images for: The utility of Escherichia coli as a contamination indicator for rural drinking water: Evidence from whole genome sequencing
Source: PLoS One. 2021 Jan 22;16(1):e0245910. doi: 10.1371/journal.pone.0245910 (PMC7822521; doi:10.1371/journal.pone.0245910)

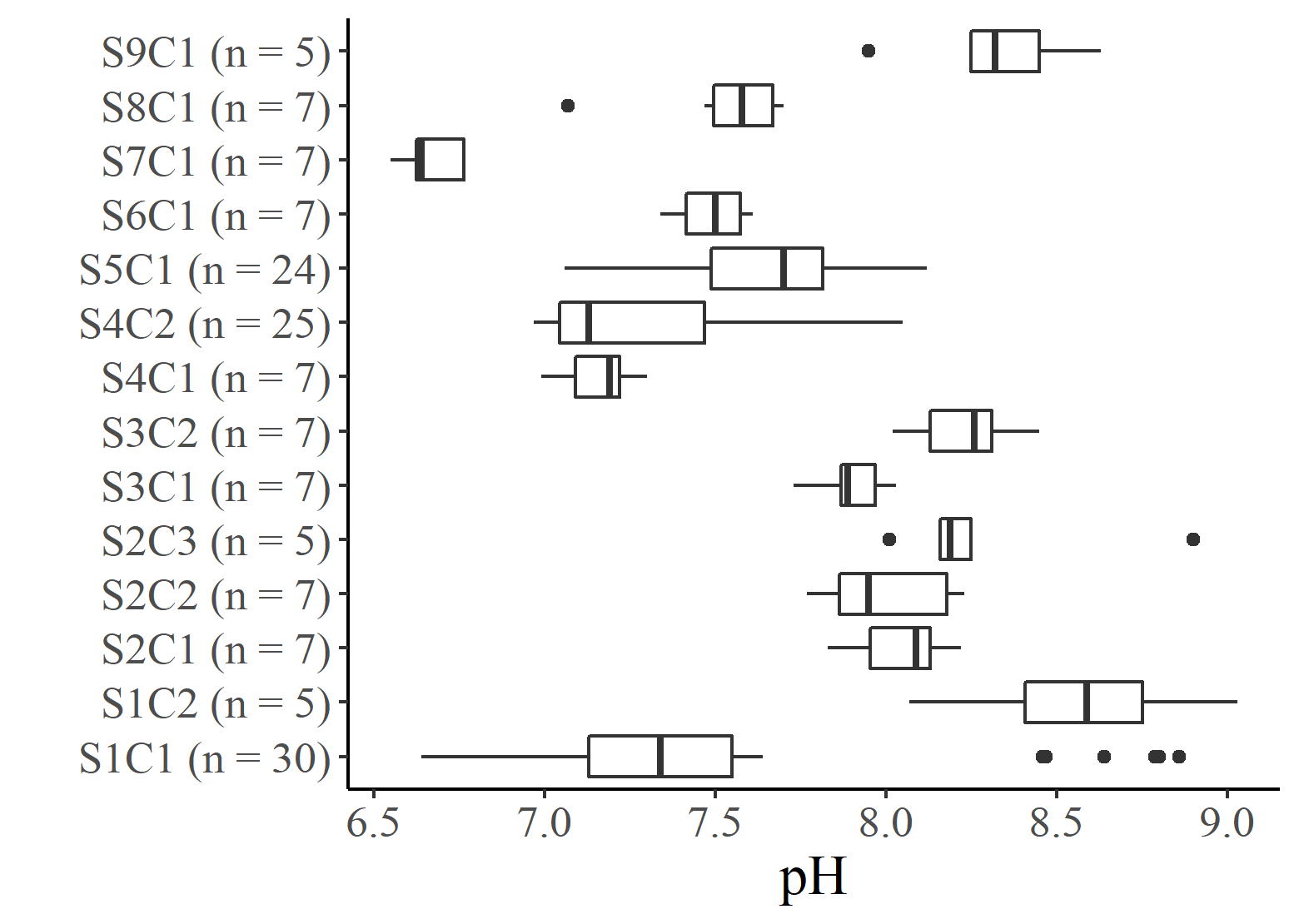

Supplement: S1 Fig — The boxes show median values and span lower to upper quartiles, the whiskers show the lowest and highest datums within 1.5 times the interquartile range. (TIFF) [file pone.0245910.s001.tiff]

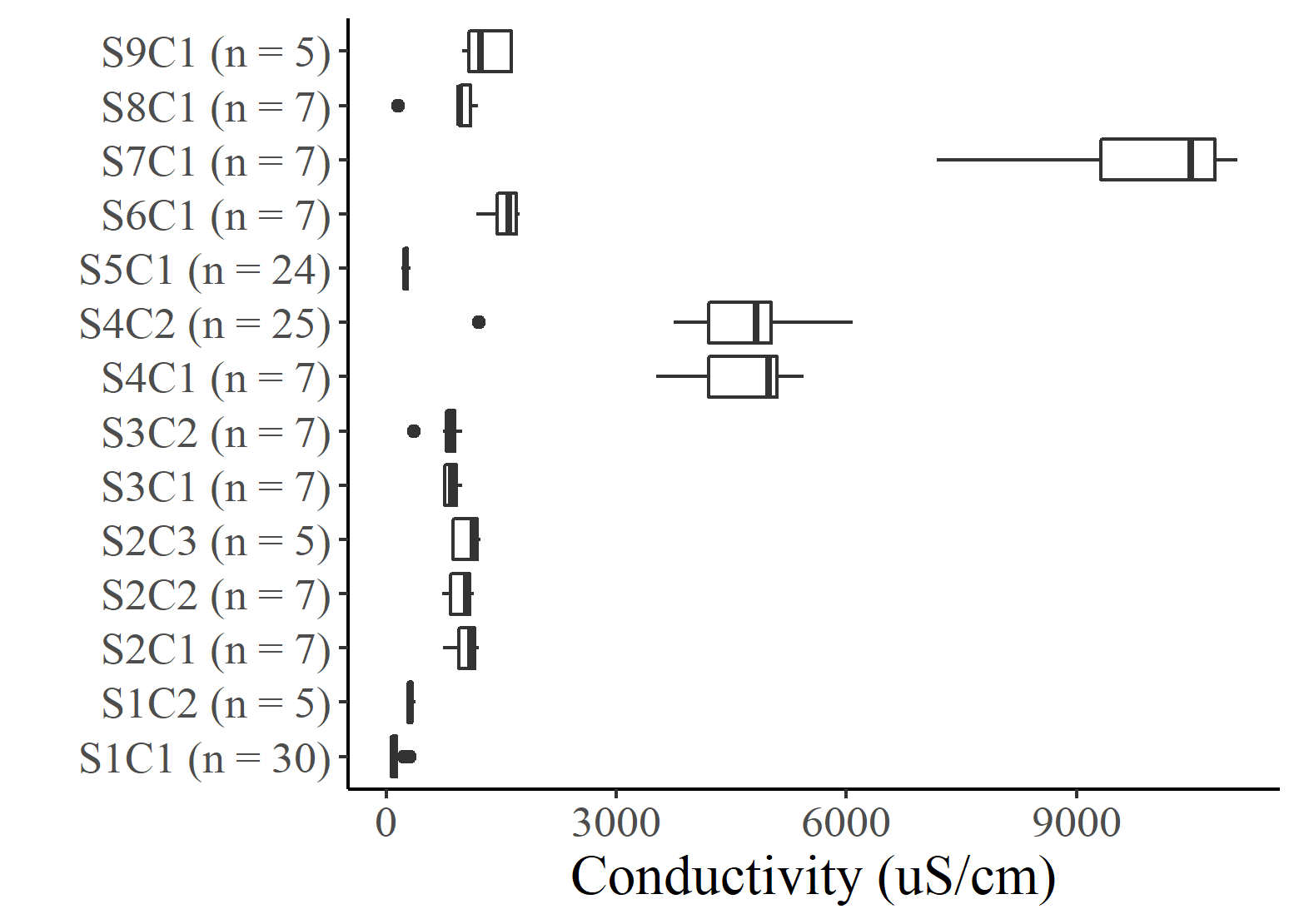

Supplement: S2 Fig — The boxes show median values and span lower to upper quartiles, the whiskers show the lowest and highest datums within 1.5 times the interquartile range. (TIFF) [file pone.0245910.s002.tiff]

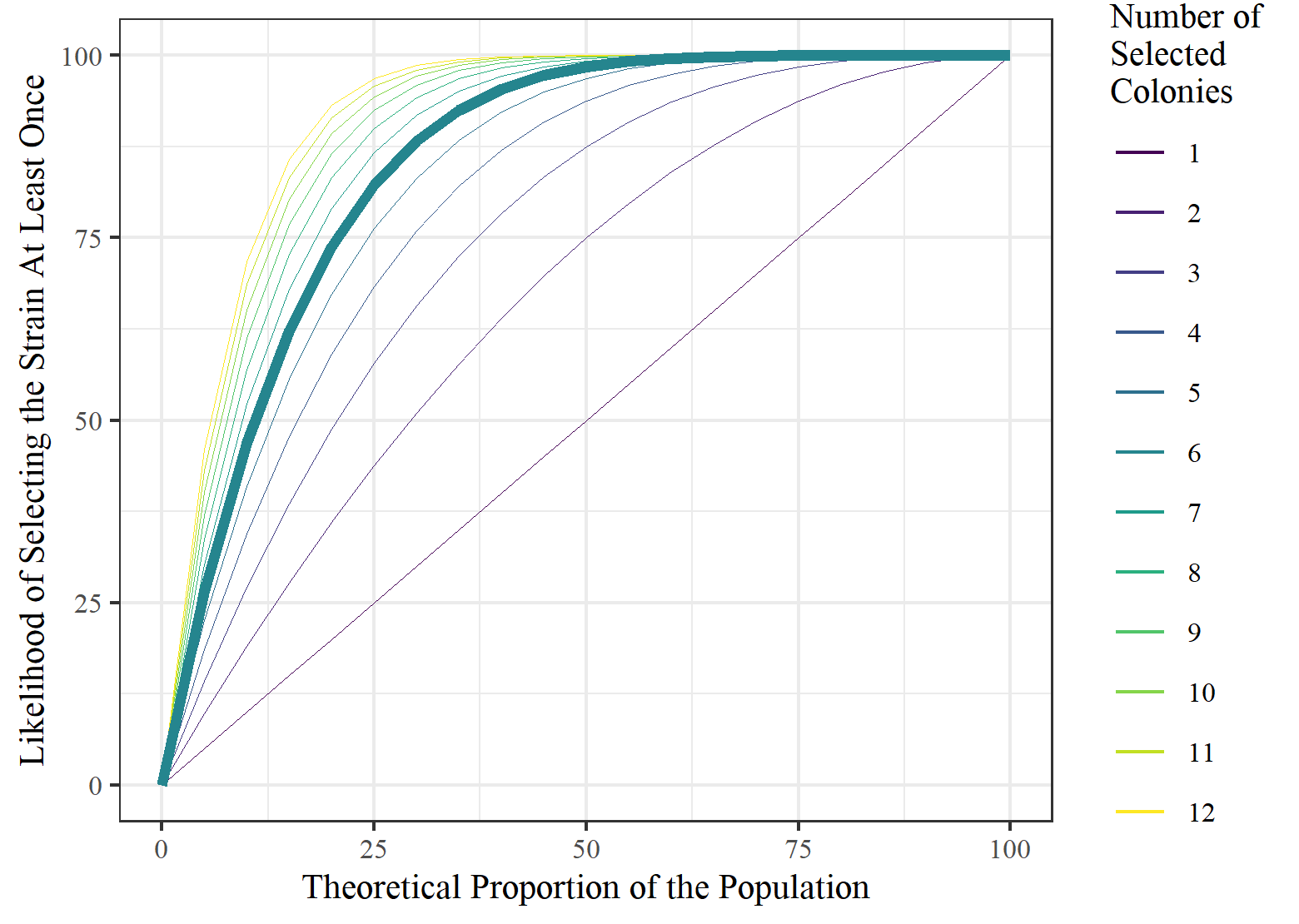

Supplement: S3 Fig — (TIFF) [file pone.0245910.s003.tiff]

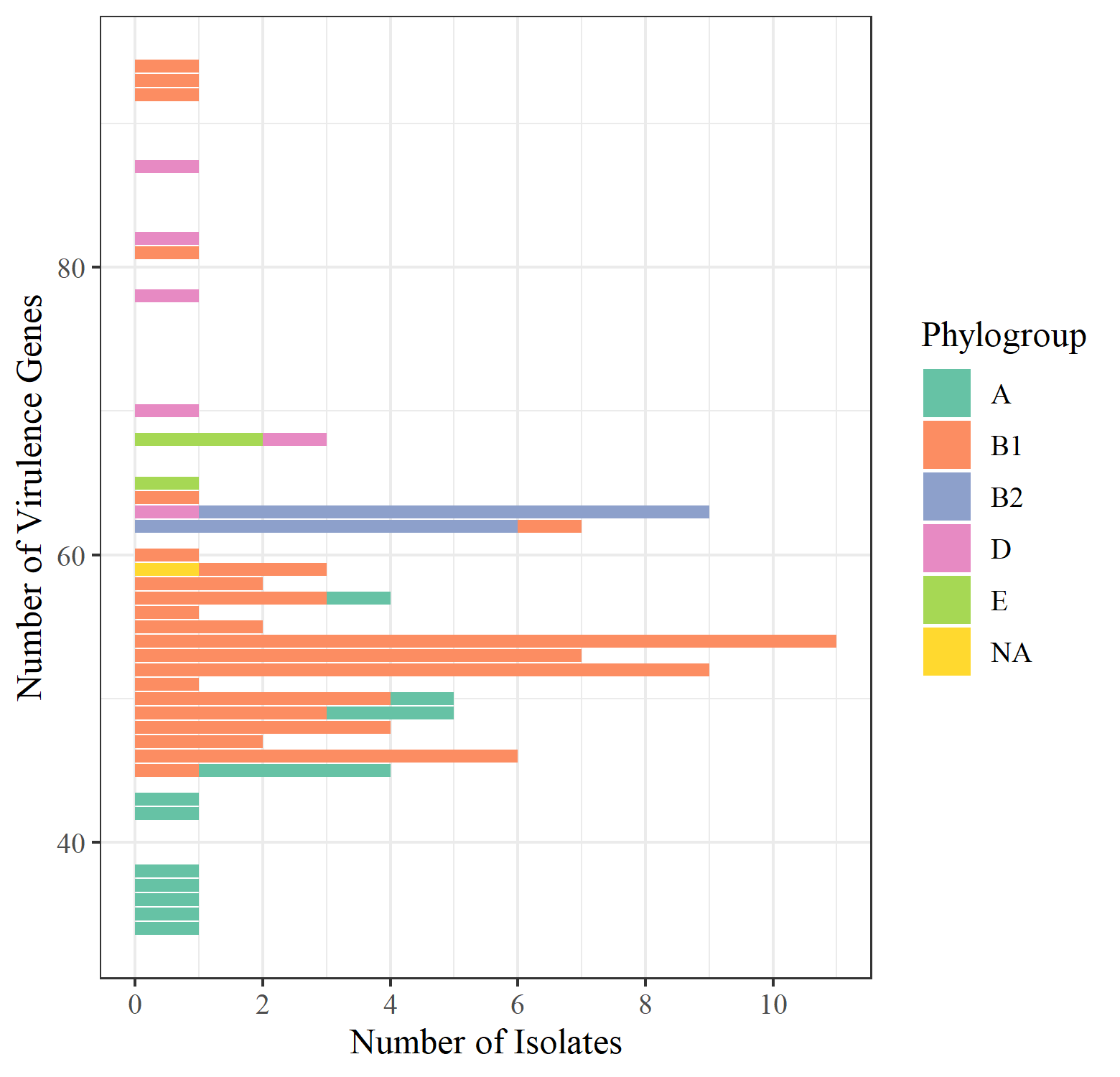

Supplement: S4 Fig — (TIFF) [file pone.0245910.s004.tiff]
